# The Compounds Responsible for the Sensory Profile in Monovarietal Virgin Olive Oils

**DOI:** 10.3390/molecules22111833

**Published:** 2017-10-27

**Authors:** Cristina Campestre, Guido Angelini, Carla Gasbarri, Franca Angerosa

**Affiliations:** 1 Department of Pharmacy, University “G. d’Annunzio” of Chieti-Pescara, via dei Vestini, 66100 Chieti, Italy; guido.angelini@unich.it (G.A.); carla.gasbarri@unich.it (C.G.); 2 Council for Agricultural Research and Economics (CREA), CREA-OLI Olive Growing and Oil Industry Research Centre, Viale Petruzzi 75, Città Sant’Angelo (PE) 65013, Italy; franca.angerosa@virgilio.it

**Keywords:** virgin olive oil, volatiles, phenolic compounds, sensory characteristics, cultivar, agronomic and technological factors

## Abstract

Monovarietal virgin olive oils (VOOs) are very effective to study relationships among sensory attributes, the compounds responsible for flavour, and factors affecting them. The stimulation of the human sensory receptors by volatile and non-volatile compounds present in monovarietal virgin olive oils gives rise to the sensory attributes that describe their peculiar delicate and fragrant flavours. The formation of these compounds is briefly illustrated and the influence of the agronomic and technological factors that affect their concentrations in the oil is examined. The relationships between compounds responsible for the olive oil flavour and sensory attributes are discussed. Several approaches for the varietal differentiation of monovarietal virgin olive oils are also overviewed.

## 1. Introduction

Virgin olive oils (VOOs) are unique among other vegetable oils. They are only mechanically extracted and can be consumed without any further refining process, thus preserving natural compounds, very important for their nutritional value, which give rise to their unique aroma and taste [1,2]. The fragrant and delicate flavour of VOOs is usually described by perceptions ascribable to fruity, the sensation reminiscent of the healthy olive fruit harvested at the optimum time of its ripeness, and by perceptions reminiscent of just cut grass, leaf, floral notes, green fruits (e.g., apple, banana, almond) or vegetables (e.g., tomato, artichoke), accompanied by more or less intense bitterness and pungency notes [3].

VOOs are rich in phenolic compounds that possess beneficial biological activities [4,5,6,7,8,9,10,11,12,13,14,15,16,17]. A significant effect in the prevention of several important diseases, all related to high concentrations of free radicals, is attributed to phenolic compounds, because of their antioxidant activity [4]. The protective activity against atherosclerosis and cardiovascular diseases [5,6,8,16,17], against several kinds of cancer [7,16] and against cognitive deficits and neuropathology is attributable to the free radical scavenging activity [9]. Phenolic compounds also contribute to the formation of oil flavour, as they are responsible for gustative and trigeminal sensory notes. Bitterness and pungency, characteristic attributes of fresh high quality oils, show the highest intensities, according to cultivar, when oils are obtained from not completely ripe fruits, and are also related to phenolic compounds.

Among the sensations experienced by assessors during virgin olive oil tasting, only bitterness, pungency and astringency are related to the stimulation of the gustative and trigeminal receptors from non-volatile compounds; the remaining manifold sensations are elicited by the stimulation of olfactory receptors by volatile compounds. This fraction, responsible for the unique aroma of VOOs, plays a very important role in human nutrition, stimulating the appetite and the gratifying effect, thus affecting the food acceptability and directing consumer preference [18,19].

## 2. Compounds Responsible for the Monovarietal VOOs Flavour

### 2.1. Phenolic Compounds

The interest in olive phenolic compounds has greatly increased recently, because of their antioxidant abilities [4,5,14,20,21], associated with both their nutraceutical properties [10,11,12,13,17,22,23,24,25], the high stability they confer to the resulting olive oil during storage [4,26,27,28,29,30,31], and their sensory impact [32,33].

Phenolic compounds in VOOs are responsible for the positive sensory attributes of bitterness and pungency [3,32,34]. The intensity of these attributes, and therefore the pleasantness of a VOO, depends on the concentration of phenolic compounds [34,35,36,37]. However, when the amount of phenols in VOOs is very high, the intensities of bitterness and/or pungency are very strong and many people with a special sensitivity to these attributes can reject this kind of oils, preferring oils characterized by pleasant green aroma and light-medium strength of bitter and pungent notes [18,19,38,39,40].

The phenolic fraction of VOOs is formed only in a little part by simple phenols, mainly hydroxytyrosol (3,4-dihydroxyphenylethanol: 3,4-DPHEA) and tyrosol (*p*-hydroxyphenylethanol: *p*-HPEA), but also caffeic acid and some hydroxybenzoic and hydroxycinnamic acid derivatives. Most of the phenolics in VOOs is represented by aglycons of secoiridoid glucosides [41,42,43,44], namely oleuropein and ligstroside, naturally occurring in the fruit and exclusively present in plants belonging to the Oleaceae family. While only trace levels of oleuropein have been detected in VOOs [45], the secoiridoid aglycons, produced during the oil extraction process as the result of the β-glucosidase activity, are partly dissolved into the oil. The most abundant are 3,4-DHPEA-EDA and *p*-HPEA-EDA, the dialdehydic forms of elenolic acid (EDA) linked to 3,4-DHPEA and *p*-HPEA respectively, and an isomer of the oleuropein aglycon (3,4-DHPEA-EA) [12,13]. An additional class of phenolics, namely the lignans, isolated and characterized in VOOs [46,47] is represented by 1-acetoxypinoresinol and pinoresinol.

Numerous studies have been carried out to clarify the relationship between taste attributes in VOOs and their phenolic compound contents: bitterness intensity was initially related to the presence of oleuropein derivatives [48,49,50], but other researchers, on the basis of sensory evaluations and amount of some phenolic compounds, have attributed the bitter sensory note to both oleuropein and ligstroside aglycons [33,51], or only to ligstroside derivatives such as *p*-HPEA-EDA [52].

In 2003 Andrewes and co-workers [32] separated the single phenolic compounds and evaluated their sensory characteristics. On the basis of the estimated taste threshold reported by the same authors, the main contribution to pungent attribute is given by the fraction containing p-HPEA-EDA, namely the deacetoxyligstroside aglycon, which elicited a strong burning pungent sensation at the back of the throat. In contrast, the fraction containing 3,4-DHPEA-EDA, at an equivalent concentration, produced only a slight burning/numbing sensation, which was perceived more on the tongue. No other phenolic fractions produced the intense burning sensation. Astringent attribute was mainly related to 3,4-DHPEA-EA, even if also other fractions contributed to elicit this sensation. Beauchamp et al. [53] isolated *p*-HPEA-EDA from different virgin olive oils, and measured the pungent intensity, confirming this compound as the principal responsible for throat irritation. Secoiridoid derivatives of oleuropein and demethyloleuropein such as 3,4-DHPEA-EDA and 3,4-DHPEA-EA have been found to be the main contributors of VOO bitterness [54]; simple phenols, lignans and flavones could not elicit any bitter sensation. 

### 2.2. Volatile Compounds

A great number of volatile compounds belonging to several chemical classes, especially carbonyl compounds, alcohols, esters and hydrocarbons, have been found in the volatile fraction of VOOs [55,56]. Lists of the different compounds, identified and assigned by GC–MS technique, were previously reported in the literature [57,58]. The C6 and C5 compounds, especially C6 linear unsaturated and saturated aldehydes, represent the most abundant fraction of volatile compounds of high quality VOOs [59,60,61]. Other compounds belong to hydrocarbons, acids, ethers, oxygenate terpenes, furan and thiophene derivatives. Some of the volatiles occurring in VOOs of different quality arise from the activities of certain microorganisms and/or moulds [62,63,64,65,66]. *Pseudomonas* and *Clostridium* genera produce five carbon branched alcohols determining the appearance of fusty defect [62]. Yeasts and/or *Acetobacter* accumulate ethanol, ethyl acetate and acetic acid, as result of their activities: quite large amounts of these compounds are responsible for the winey/vinegary defect [65]; butyrates and 2-ethyl butyrates, related to muddy sediment off-flavour, are formed by butyric fermentation of micro-organisms belonging to the *Clostridium* genus [57]. Numerous *Aspergillus* and *Penicillium* genera are involved in the production of C8 primary and secondary alcohols and C8 ketones, responsible for musty defect [63]. Furthermore, the accumulation of products arising from the hydroperoxides fragmentation in oils that have suffered an oxidizing process is responsible for rancid sensory defect [61].

**Figure 1:**
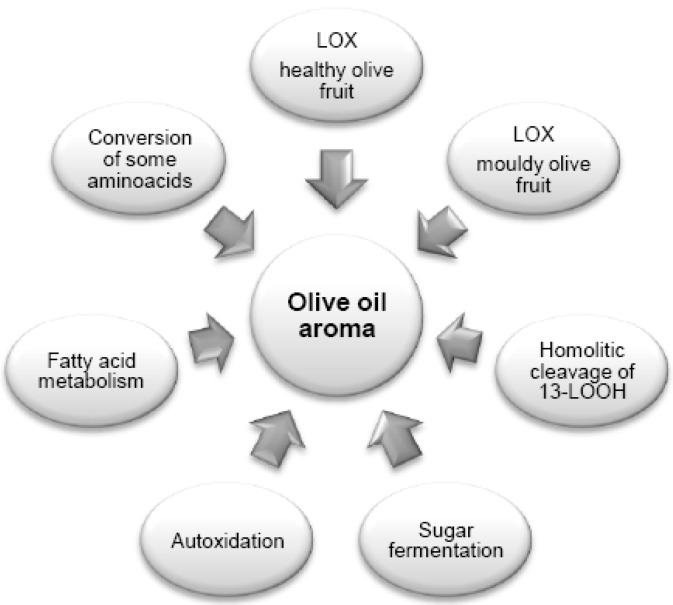
The main pathways involved in the formation of volatile compounds in VOOs.

Several pathways contribute to the production of volatile compounds of VOOs and the different sensory nuances that can be appreciated depend on the relative importance of each pathway (Figure 1).

The level and the activity of the different enzymes that are involved in the pathways affect the qualitative and quantitative composition of the volatile fraction; their content is regulated by genetic factors, whereas their activity is modulated by agronomic and technological variables [55,57,67]. Newly formed compounds, deriving from phenomena of degradation, fermentation and autoxidation, are added to compounds coming from the pathways typical of fruits. Some of them are active in olive fruit, other ones during oil extraction.

In high quality VOOs, the contribution of pathways involving activities of microorganisms or autoxidation process is practically negligible, and therefore only the lipoxygenase (LOX), the homolytic cleavage of hydroperoxides (13-LOOH) pathways and the conversion of leucine, valine and isoleucine are really active [57,68]; among them, because of the considerable amounts of corresponding metabolites, the major activity is displayed by LOX pathway.

Most of volatiles are rapidly formed during the olive crushing, owing to the disruption of olive cells [69]. Their production also continues during the malaxation step of olive processing. 

**Figure 2:**
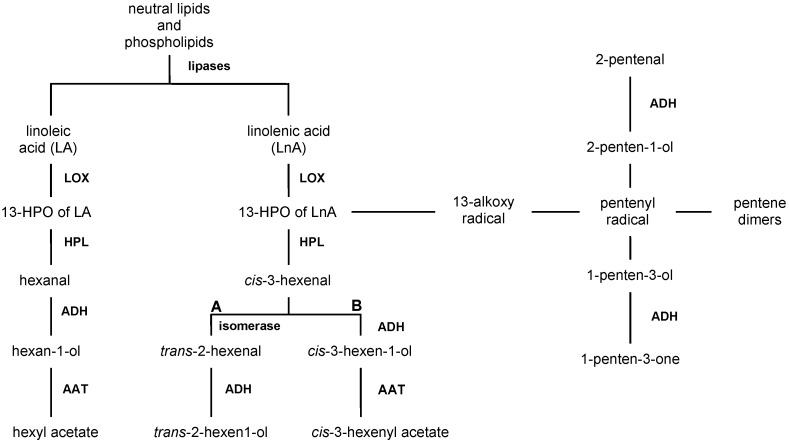
LOX pathway of volatile compounds in VOOs.

Volatiles are biosynthesized in plants as a response to cell disruption from C18 unsaturated fatty acids containing a *cis*,*cis*-1,4-pentadiene structure, namely linolenic (LnA) and linoleic (LA) acids, by a LOX oxidation [59,60,61]. The pathway starts from the oxidation of linolenic (LnA) and linoleic (LA) acids mediated by LOXs, and a number of other enzymatic activities lead to the accumulation of C6 volatile compounds (Figure 2). 

In addition C10 hydrocarbons (also known as pentene dimers) and C5 alcohols, namely 2-penten-1-ol and 1-penten-3-ol, would be produced from the LnA hydroperoxide through an additional pathway, partly enzymatic, that involves an alkoxyl radical; the subsequent oxidation of C5 alcohols could lead to C5 carbonyl compounds [70].

The LOX pathway involves, in addition to the activity of LOX [71], in sequence those of hydroperoxide lyases (HPL) [72], alcohol dehydrogenases (ADH) [73], and alcohol acetyl transferases (AAT) [74], whose levels are genetically determined, so that, according to their individual content and activity, there is a different accumulation of C6 and C5 compounds, accounting for the various sensory profiles of VOOs.

It is very difficult to relate each volatile to sensory perceptions. HRGC-sniffing techniques were applied by several researchers to investigate about the odour quality of compounds present in the oil aroma [58,75]. Odour qualities of volatile compounds found by different authors and corresponding references were reported in a paper [55]. Odour intensity seems to be more linked to a series of chemical factors (e.g., size, volatility, type and position of functional groups) than to concentration [75,76,77]. Differences in individual sensitivity of human subjects affect the evaluation of the odour intensity [78], so that the contribution of each volatiles to the oil aroma is better evaluated by the odour activity value (OAV), that is the ratio between the concentration and the corresponding odour threshold [79,80]. According to Guth and Grosh [79,80] the most important contributors in VOOs high quality are *cis*-3-hexenal, hexanal and *cis*-3-hexen-1-ol, because of their low odour threshold.

However the aroma of VOOs is the result of complex interactions occurring between volatiles and receptors responsible for taste, smell, sight and trigeminal and tactile perceptions [35,76,81,82]. Thus positive and negative synergisms can occur and new kinds of perceptions could be produced by the interaction between taste and odour [82]. In spite of these interactions, in literature sensory perceptions are related to the concentrations of volatile compounds. Principal component analysis (PCA) and partial least square regression (PLS) analysis were used to relate sensory and instrumental data by Servili and co-workers [83]. PLS regression gave good predictions from headspace data of some of the descriptors used in quantitative descriptive analysis. Inter-intra dissimilarities from data sets of sensory attributes and volatile components were studied by means of multidimensional scaling (MDS) [84]: volatiles completely explain the sensory perceptions, as described by different panels and categorized by them into aroma, odor, taste, after-taste, mouthfeel and after-mouthfeel. The plot of the first two dimensions of MDS of datasets of volatiles and sensory notes evidenced the presence of seven basic sectors (green, fruity, sweet, ripe, over-ripe, undesirable, and pungent) and volatiles responsible for them. These results were in agreement with conclusions achieved by using a sensory wheel [38,85,86], a robust statistical procedure that allowed clustering into the same seven sectors sensory attributes with the same semantic description, obtained from a panel of six different countries. The position of descriptors on the sensory wheel was found consistent with the sensation elicited by the pure compound tested by HRGC sniffing methodology. The relation between sensory and instrumental data was determined by projecting volatiles onto the sensory wheel. Volatiles took up the appropriate place within the sector that corresponded to their perceptions detected by sniffing method: all *cis*-3 compounds from LOX pathway belonged to the green sector, hexanal was placed in the sweet one and some compounds such as *trans-*2-hexen-1-ol and hexan-1-ol in the undesirable sector.

Angerosa et al. [35] applied a Linear Regression Analysis (LRA) to sensory attributes perceived by panel tasters and to C5 and C6 compounds from LOX pathways and phenolic compounds. Results evidenced that hexanal plays an essential role in the formation of the majority of green attributes: this compound showed a positive correlation with sweet sensory note, and a negative one with grass and leaf sensations. *trans*-2-Hexenal contributed to grass perception, and 1-penten-3-one together with phenolic compounds to leaf attribute. 1-Penten-3-one and *cis*-3-hexen-1-ol seemed to have a synergic effect on the elicitation of bitter and pungent characters, positively correlated with phenolic compounds and negatively with hexanal. Phenolic compounds contributed to the characterization of walnut husk, whereas C5 compounds, especially 1-penten-3-one, strongly affected most attributes (Table 1).

## 3. Factors Affecting Compounds Responsible for Flavour of Monovarietal VOOs

While enzyme levels are genetic characteristics and therefore typical of the cultivar, their activity is affected by several but not less important factors, related to ripeness and growing area of fruits, time and conditions of their storage, technological aspects of oil extraction. Therefore these parameters quantitatively modify the composition of flavour compounds. Monovarietal oils are very useful for studying the influence of factors such as agronomic or technological ones on the sensory notes and on compounds responsible for their flavour.

### 3.1. Agronomic Factors

#### 3.1.1. Cultivar

Cultivar plays an essential role, as the amount of the enzymes involved in the pathways of volatile and phenolic compounds is genetically determined. The phenolic composition of olive fruit is tightly connected to genotype [87,88,89,90]. 

Oleuropein and demethyloleuropein are the predominant secoiridoids of olive fruit, which also contains verbascoside. The phenolic composition of olive fruits from different cultivars is also reflected on that of corresponding oils. While the phenolic profile is almost the same, the absolute concentration of each secoiridoid derivative is cultivar dependent [12]. The concentration of the lignans 1-acetoxypinoresinol and 1-pinoresinol in VOOs is strongly related to the botanical origin, and could be used as varietal index [12].

The influence of the cultivar on the volatile fraction can be evidenced by the different concentrations of these compounds if changes of the activity of enzymes involved in the LOX pathway are removed. Table 2 reports the concentrations of C6 and C5 compounds in oils, obtained in the same operative conditions of the extraction process, from fruits of different cultivars grown in the same botanical garden and harvested at the same ripening stage.

**Figure 3:**
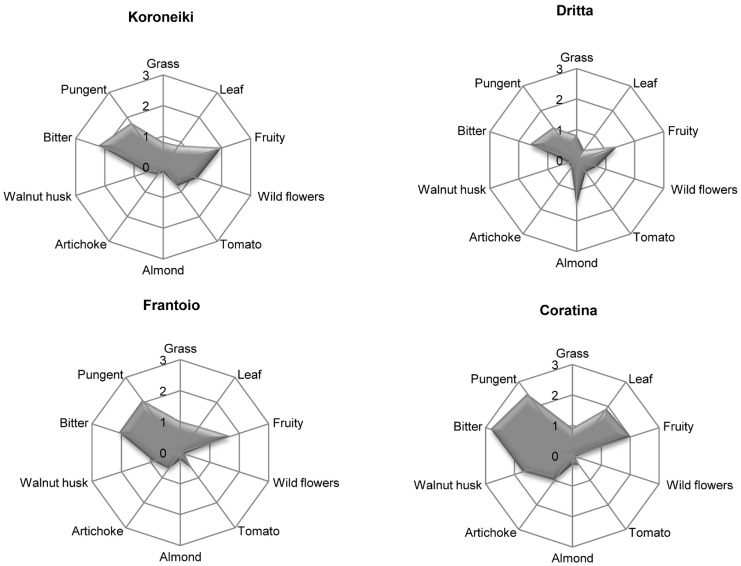
Sensory profiles of extra virgin monovarietal oils from Koroneiki, Dritta, Frantoio and Coratina cultivars. (Source: Authors; unpublished results).

The different genetic stores give rise to a different total amount of compounds from LOX pathways. *trans*-2-Hexenal is the most abundant compound in all the seven cultivars, but its concentration is quite different: Frantoio and Canino show the higher values, while Koroneiki and Moraiolo the lower ones. Similar contents are observed for Dritta and Bosana. These results agree with the findings of Aparicio and Luna [91], who suggested that monovarietal VOOs could be distinguished by *trans*-2-hexenal content. Coratina and Canino have a negligible concentration in esters, which, on the contrary, characterizes Frantoio, Bosana and especially Koroneiki. Also the ratio between total amounts of C6 compounds from LnA and LA respectively and the one between total amounts of C6 compounds from LnA and total amount of C5 compounds are different and evidence varietal differences. Several investigations correlated C6 and C5 compounds with green perceptions [35,61,86,92], thus the different concentration of C6 and C5 compounds will elicit the different nuances and intensities of green attributes. Figure 3 shows the sensory profiles of some of the oils reported in Table 2.

#### 3.1.2. Ripening Degree

The development and the ripening of olive fruit are a combination of biochemical and physiological events that occur under both strict genetic control and influence of several environmental conditions [93]. It is generally accepted that the content of glucosides in the fruits, and consequently that of aglycons in the oil, shows a first reduction in the green ripening phase because of an increased activity of hydrolytic enzymes; it then falls after fruits turn to purple colour [94,95,96,97].

An esterase activity has been invoked to explain the rapid decline of oleuropein in the black ripening phase [94]. Consequently to the hydrolytic activity, the amount of simple phenols increases during the ripening process [88,95,98,99,100,101]. The presence of high levels of phenolics enhances the nutritional and healthy properties of the resulting oils, but it is related to strong intensities of bitter and pungent, not very appreciated by habitual and potential consumers [18,19,40].

**Figure 4:**
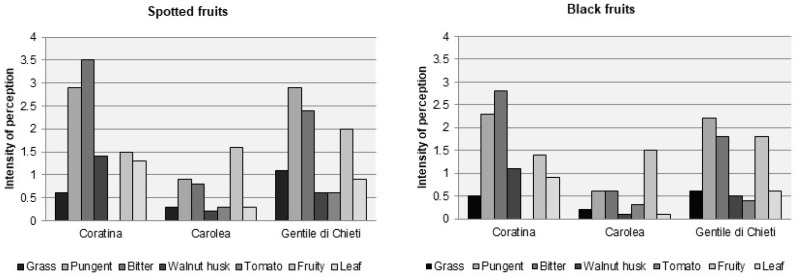
Sensory profiles of oils from Coratina, Carolea and Gentile di Chieti cultivars, at two different stages of ripeness. Figures were obtained on the basis of data collected by our team. Data represent the average of intensities recorded by fully trained tasters in three independent trials. The oils were presented according to an experimental design, which minimized possible biases and carry-over effects. Standard deviation ranged between ±0.2–0.5. (Source: Authors; unpublished results).

The significant decrease of phenolic fraction, especially of oleuropein from spotted until black fruits, is perceived as a weakening of bitterness and pungency in VOOs [32,54,55]. The weakening does not only concern bitter and pungent attributes, but also the olfactory perceptions; this means that also volatile compound profile changes, according to the progress of ripeness (Figure 4).

The evolution of the volatiles responsible for VOO green aroma in relation to ripening degree of fruits was studied by several researchers. Some of them [102,103,104], studying monovarietal oils extracted with the same plant and conditions from fruits grown in the same botanical garden, found that, during the ripening, the amount of volatiles, especially C6 aldehydes, increases up to a maximum concentration occurring at spotted or reddish ripening stage; beyond that point, the volatile content decreases, because of the activity decline of enzymes involved in their production, with a consequent weakening of the intensity of some “green” sensory notes, as depicted in Figure 4 [102,103,104,105,106]. The content of volatile compounds and the stage at which the maximum is achieved are variety-dependent. Gómez-Rico et al. [100] confirmed an increase of C6 compounds in the early stages of ripeness in some Spanish cultivars. Aparicio and Morales [105] described a steady decrease of the level of the volatile compounds, including *trans*-2-hexenal, from the unripe to the over-ripe stages, except for oil from Coratina fruits that, on the contrary, showed the behaviour observed by the other researchers [102,103,104]. Angerosa and Basti [102] evidenced the decrease of C6 esters and alcohols from LnA and the increase of hexan-1-ol, a compound considered eliciting a not completely desirable odour [85,86] in the later ripening stages.

The application of multiple regression to the quantitative values of volatile compounds allowed Aparicio and Morales [105] to conclude that the major contributors to the characterization of the ripening process are *trans*-3-hexen-1-ol, *cis*-3-hexen-1-ol, *trans*-2-hexen-1-ol, hexanal and hexyl acetate. The best characterized ripening stage was the unripe one.

#### 3.1.3. Pedoclimatic Factors

In the last thirty years many cultivars has been extensively cultivated outside their traditional area, in new producer zones where they were not present at all. This is the case of some varieties that are now cultivated either in areas of the same country where they were not autochthonous (see Northern cv Arbequina in the South of the Spain), or even in countries where olive tree was completely unknown (see Frantoio and Leccino cv in Andalusia, Tunisia, Argentina, South Africa). The use of non-autochthonous cultivars is interesting to evaluate their productivity and adaptation capacity to different agronomical conditions, together with the composition and sensory characteristics of the resulting oils. Temperature, water availability and altitude often are very different from those of the habitual growing areas, and several researches were carried out to elucidate their influence on the physical-chemical and sensory composition of the oils.

The majority of these investigations were carried out on monovarietal oils: in these studies the ripening stage of fruits and the conditions of oil extraction were made uniform to evidence the influence of environmental factors.

• Altitude

Phenolic and volatile compositions are affected by altitude and temperature since these factors modify the enzymatic activities. Issaoui et al. [107] evidenced higher levels of *trans*-2-hexenal and 1-hexanol in oils from low altitude and high temperature. The greater abundance of *cis*-3-hexenyl acetate and hexyl acetate, the first associated to green odors and the last described as fruity, pear-like by Bauer et al. [108], seem to characterize the oils from high altitude and low temperature. These results are consistent with those of Vichi et al. [109], who found that levels of hexanal, 1-hexanol, *trans*-2-hexenal, *cis*-3-hexenal, *cis*-3-hexen-l-ol, *trans*-3-hexen-l-ol, and *trans*-2-hexen-l-ol showed a strong dependence on geographical origin. 

Pedoclimatic conditions seem to influence the contents of some hydrocarbons, that have been indicate as possible markers for the varietal or geographical origin characterization [91,109,110,111]. In oils from mountain the levels of α-copaene, α-pinene and *trans*-β-ocimene were significantly higher than in oils from low altitude and high temperature [107,109,112]. Several studies [107,113] showed a higher total amount of phenolic compounds and o-diphenols in oils from high altitude and low temperature, compared with those from low altitude and high temperature. The changed composition in relation to different environmental conditions affects the sensorial characteristics: oils from mountain elicited more intense notes of fruity and bitterness and pungency than those from fruits grown at sea level [107]; sometimes assessors perceived wood and fig tree attributes, typical of oils with high phenolic concentration in oils from high altitude [113].

• Water stress and irrigation

Olive trees come from regions with limited water resources and are generally grown under rain-fed conditions, but in the last years many new orchards have adopted irrigation techniques to considerably increase fruit yields per hectare and therefore total production of the oil [114,115]. Results of an investigation carried out in the climatic conditions of Central Italy gave evidence that the changes in volatile composition due to rainfall were pre-eminent and the rainfall were negatively correlated to some compounds, such as hexanal and isobutyl acetate [116]. These features were confirmed by Gómez-Rico et al. [117], who studied the modification of volatile fraction in oils from Cornicabra and Morisca cultivars grown under different irrigation strategies. They found that in both cultivars, the volatiles mostly affected by the water status of olive trees were hexanal, *trans*-2-hexenal and hexan-1-ol, which show an inverse relationship with the water stress integral observed in the plants. The concentration of VOOs volatile compounds from a medium–high stressed treatment was clearly lower than that obtained under irrigation strategies. Similar trends in C6 aldehydes and alcohols were reported by other researchers in oils from Cornicabra [118] and Leccino [119] cultivars.

On the other hand, Aparicio and Luna [91] and Gómez-Rico and co-workers [118] evidenced differences in the oil composition and in some sensory characteristics of VOOs from irrigated and rain-fed olive trees. In particular they found a greater phenolic concentration and more intense bitterness and pungency in oils from fruits of rain-fed trees than in those from irrigated trees. A similar behaviour was observed also by other researchers [120,121,122,123]. The different total amount of phenolic compounds has been related to the water stress level of olives from rain-fed to irrigation conditions, since the activity of enzymes responsible for phenolic compound synthesis, such as l-phenylalanine ammonia-lyase, is greater under higher water stress conditions [121,124].

The water availability affects not only the total amount of phenolic compounds, but also their composition. In oils from some cultivars, such as Arbequina, Cornicabra, Morisca, Leccino, a great decrease in 3,4-DHPEA-EDA, 3,4-DHPEA-EA and *p*-HPEA-EDA was found, as the water stress of olive trees fall off [52,112,118,119,125,126,127], probably for a reduced activity of enzymes involved in the phenolics synthesis [121], due to the irrigation. As a consequence of the decrease of 3,4-DHPEA and *p*-HPEA secoiridoid derivatives [118], water volumes affect the oxidative stability [128] and the intensities of bitterness and pungency perceptions, being both tightly connected with the concentrations of phenolics [4,32,54,129]. 

A considerable weakening of these sensory notes in oils from irrigated olive trees was observed by several researchers [117,123,130], but their decline in these oils was also accompanied by the weakening of odour notes [117]. Low water levels produced oils that emphasize woody and herbaceous sensory notes and very strong intensities of bitterness and pungency that make these oils poorly attractive for consumers; conversely high irrigation levels lowered oil aroma and considerably reduced bitterness and pungency [123,125,127,130,131].

Medium irrigation levels produced complex oils characterized by several well balanced attributes such as artichoke, grass, green apple, and pleasant intensities of bitter and pungent notes [130]. Therefore, the suitable control of the irrigation levels could be a useful tool to produce oils well appreciated by consumer, being characterized by agreeable intensities of olfactory notes, bitterness and pungency. These findings are of great interest, especially for cultivars characterized by high concentrations of phenolic compounds, such as the Spanish Cornicabra and the Italian Coratina varieties. It should be recommended to grow these cultivars under irrigation regime, since high levels of phenolics may negatively influence the olive oil acceptability. The concentrations of lignans 1-acetoxypinoresinol and pinoresinol are slightly influenced by the irrigation practices and should seem to decrease in oils extracted from fruits of low irrigated treatments [119,125,127].

#### 3.1.4. Time and Conditions of Preservation of Fruits

In high quality VOOs, obtained from fruits immediately processed after their harvesting, there is not time for establishing phenomena of degradation and/or fermentation and no effects are observed on the volatile and phenolic composition. Irrational conditions and relatively long times of the olive preservation determine both the decrease of the concentration of volatiles from the LOX pathways and phenolic compounds, and the production of volatile compounds related to off-flavours [62,63,64,65,66], as described above.

### 3.2. Technological Factors

The quality of virgin olive oils is tightly dependent on the process of their extraction from olive pastes, where the enzymatic activities represent the main cause of modification of their both sensory characteristics and antioxidant store. On the basis of the system and the conditions adopted for oil extraction, the various mechanical actions cause, with a different importance, a series of interactions among oily phase, vegetation water and solids in the olive paste. This results in: (1) incomplete recovery of the oil that partly remains into pomaces and partly goes away in the vegetation water; (2) losses of minor compounds; (3) beginning of hydrolytic and oxidative phenomena, which can compromise the oil quality. All the steps of oil extraction can modify the volatile and phenolic fractions: the more critical steps are represented by olive crushing and paste malaxation.

#### 3.2.1. Crushing

Olive crushing is performed with stone mill or with hammer crusher. During crushing, when the oil flow from vacuole owing to the cell disruption, several enzymes are activated: their activities result in the hydrolysis of secoiridoid glucosides, with production of corresponding aglycons, and in the activation of the LOX pathways that rapidly give rise to C6 and C5 compounds. 

The concentration of phenolic compounds in olive oils is dependent on the activities of native enzymes occurring in the fruits that mainly affect the technological steps of crushing and malaxation [3,55,132,133]. The secoiridoid glucosides of olive fruits are hydrophilic compounds and they are not solved into the oil during the extraction process. However, the activation of the endogenous β-glucosidases during crushing [34,134] allows the production of 3,4-DHPEA-EDA, 3,4-DHPEA-EA and *p*-HPEA-EDA in olive oils; in fact the inactivation of these enzymes in blanched olives before crushing is responsible of the absence of aglycons [34] in the corresponding oils.

While the kind of metallic crusher (hammer or blade crusher) poorly affects the amount and the composition of phenolic compounds, as reported by Servili et al. [133], some difference was found in oils obtained with stone mill or with metallic crusher [51]: oils from metallic crusher were more bitter and pungent than those from stone mill. Conversely, the kind of crusher affects the amount of volatile compounds [51] formed during this technological operation as a response to cell disruption. The violence of grinding that characterizes the action of metallic crushers, greater than the one of stone mills, causes an instantaneous rise of the temperature that reduces the activity of HPL. This enzyme, catalyzing the fragmentation of hydroperoxides, plays a crucial role in the formation of volatiles. Its activity has a maximum at temperatures around 15 °C [72]; beyond this temperature its activity rapidly declines and therefore a lower amount of volatiles is formed when compared with that observed in oils from more soft crushing. 

Salas et al. [67], evaluating the impact of the depletion of LOX and HPL on the volatile composition of leaves from potato plants, evidenced a severe decrease in the amount of volatiles produced by the leaves and in the intensity of their aroma, confirming the crucial role played by HPL in the production of C6 compounds responsible for green attributes.

Angerosa and Di Giacinto [51] compared volatile fraction of oils obtained by using stone mill and disc metallic crusher. They concluded that a greater accumulation of hexanal, *trans*-2-hexenal, *cis*-3-hexen-1-ol is observed by grinding olives with traditional stone mills. In addition oils from stone mill were more aromatic and balanced than those obtained by using the disc crusher. Data are in agreement with results of Servili and co-workers [133]: oils obtained by using the blade crusher show significant increases of C6 aldehydes and esters and reduction of some alcohols such as 1-hexanol and *trans*-2-hexen-1-ol with respect to the oil obtained by using the hammer crusher.

**Figure 5:**
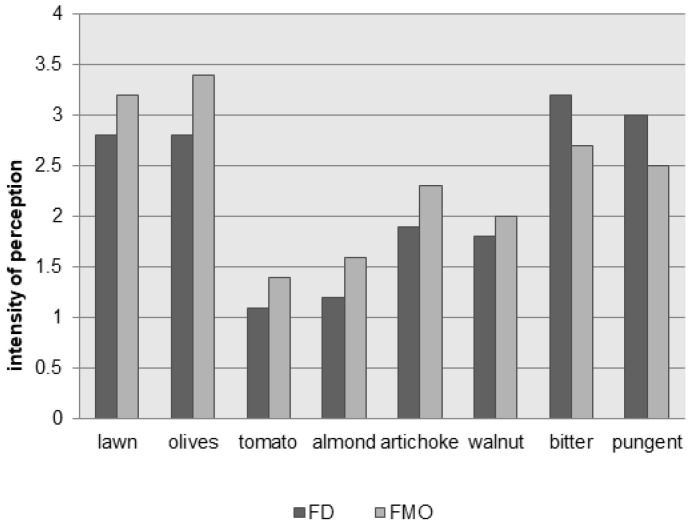
Sensory profiles of oils from fruits of two batches of Provenzale cultivar obtained with the same processing diagram except for crushing. FD = hammer crusher; FMO = stone mill. Figures were obtained on the basis of data collected by our team. Data represent the average of intensities recorded by fully trained tasters in three independent trials. The oils were presented according to an experimental design, which minimized possible biases and carry-over effects. Standard deviation ranged between ±0.2–0.5. (Source: Authors; unpublished results).

Hexanal and *trans*-2-hexenal are correlated with apple, green, sweet and just cut grass, bitter almond respectively, hexyl acetate and *cis*-3-hexenyl acetate with sweet, fruity and green banana, green leaves respectively [49], whereas 1-hexanol and *trans*-2-hexen-1-ol are related to hay-like sensory note [135]. The sensory analysis (Figure 5) confirmed that the violence of crushing has a negative impact on the intensities and on the harmony of sensory notes [51,133].

• Destoning

Destoning is a new non-conventional olive processing system by which removal of stones before kneading the olive paste increases the quality and sensory properties of VOOs. This improvement arises from the different composition and distribution of endogenous enzymes in olive fruit [34,136]: the partial inhibition of peroxidase (POD) activity, mainly contained in the endosperm of olive, reduces the oxidative degradation of hydrophilic phenols during processing, and increases their content in VVOs. On the other hand destoning does not affect the activity of LOX, mainly contained in the olive pulp tissues, so that the production of volatiles is assured. Removal of the olive stone evidences in the corresponding oils a considerable increases of the phenolic fraction, especially the secoiridoid derivatives such as 3,4-DHPEA-EDA, *p*-HPEA-EDA and 3,4-DHPEA-EA, whereas no significant variations of lignans are observed. Moreover destoning also modifies the volatile profile of VOOs. In oils obtained from destoned olives there is an accumulation of C6 compounds, mainly those arising from LA, compared with oils extracted from whole fruit [136,137,138]. The high quantity of C6 compounds may be explained by a greater release of membrane-bound enzymes involved in the LOX pathways, owing to a higher degree of cellular damage caused by the grinding of pulp tissue in destoned fruits [137]. Hexanal, reminiscent of green apple or green fruit odour notes [105], gives a great contribution to olive oil flavour because of its low odour threshold [80], and plays an essential role in the formation of majority of green attributes [35].

#### 3.2.2. Malaxation

Malaxation consists in a low and continuous kneading of the olive paste and it is an essential operation for achieving high and satisfactory yields of extraction, expecially if the crushing has been performed by using a metallic crusher that causes the oil breakdown into very small droplets. The slow kneading of the olive paste causes the repeated breakdown of lipoproteic membranes, improving the coalescence of the oil droplets, and allows the partition of compounds between the oil and watery phase and vice versa. In the same time, the active endogenous enzymes produce secoiridoid aglycons from corresponding glucosides, oxidized phenolics and volatiles, generating compounds responsible of the flavour. 

During malaxation, glucosides degradation gives rise to a significant decrease of their content [139]. The observed losses of secoiridoids derivatives are related to both the greater solubility of phenolic compounds into the aqueous phase [140] and to enzymatic oxidative processes, mediated by polyphenoloxidases (PPO) and peroxidases (POD) [141] occurring in the olive paste. In addition, some interactions between polysaccharides and phenolic compounds may reduce the release of these latter into the oil, contributing to justify the losses due to malaxation step [142].

Time and temperature of processing and oxygen concentration affect the level of phenolic compounds [130,143,144]. High temperature more than time of malaxation causes greater losses in phenolic compounds, because of an increase of PPO and POD activities [132,143,145]. The losses of the phenolic fraction have important repercussions, besides on the gustative perceptions, on the oil stability against the oxidation, and therefore on its shelf-life [146].

Malaxation time and temperature should be attentively studied for the oil production from cultivars especially rich in phenolic substances, such as the Italian Coratina and the Spanish Cornicabra, in order to reduce the concentration of phenolic substances without modifying in a considerable way the volatile compound level; the suitable choice of these parameters will weaken the intensity of bitter and pungent attributes, making the oil more agreeable for consumers.

On the contrary, it is important to preserve phenolic compounds in the production of oils from cultivars having a low phenolic content. A partial inhibition of PPO and POD, performed by reducing the O_2_ level in the paste, that is replacing air with N_2_ in the headspace of malaxer during processing, minimizes the oxidative degradation of phenolic compounds determining the strong increase of their content in the oil [34,139,147,148] with significant repercussion on its sensory and healthy qualities. A research carried out modifying the Time of Exposure of Olive Paste to Air Contact (TEOPAC), with the aim to optimize the malaxation operative conditions in the extraction of Frantoio and Moraiolo oils, evidenced the deleterious effect of high temperatures on the oil quality, according to results of other researchers [132,143,149,150].

Higher amount of phenolic compounds are detected in oils from malaxed destoned pastes than those of oils from integral ones. This feature is explained by a reduced activity of peroxidases (POD), partially eliminated being mainly contained in the olive seed [151]. Moreover this increase could be also dependent on the greater extraction of phenolic compounds due to minute shapes of the olive paste. Volatile compounds, in addition to phenolic ones, are affected by malaxation, because of the activities of enzymes of LOX pathways during the kneading of the olive paste. 

Consumers like almost all aroma descriptors qualified with the adjective green [18,19] and therefore the presence of volatiles responsible for pleasant sensory perceptions should be promoted. The ratio among volatiles in the final product is only modified by changing temperature and time of malaxation. Prolonged times increase yields, but promote the accumulation of C6 alcohols and aldehydes, especially hexanal. But long times, in addition to the reduction of phenolic compounds, cause the loss of C6 esters and the increase of C6 alcohols [143], included hexan-1-ol and *trans*-2-hexen-1-ol, related to not completely agreeable perceptions [86]. However the malaxation temperature has the most important effect on the olive oil volatile composition. High temperatures, in addition to the loss of phenolic compounds, have as a consequence the reduction of the amount of all volatile compounds, especially of *cis*-3-hexen-1-ol and C6 esters, because of the partial inactivation of HPL [150]. Moreover high temperatures promote the production of *trans*-2-hexen-1-ol, a volatile characterized by a green smelling and an astringent bitter tasting, the latter being an undesirable sensory perception for potential consumers [18].

Prolonged times, and more heavily, high temperatures cause the increase of branched aldehydes, quite low in high quality VOOs, but without accumulation of corresponding alcohols [143] correlated with “fusty” defect [62,66].

In general, the highest concentrations of aldehydes are measured when malaxing time is shorter, the production of alcohols is promoted at high temperature (35 °C), and the concentration of esters is higher at low temperature (25 °C). Malaxing at high temperatures (≥35 °C) promoted formation of green volatiles responsible for undesirable sensory perceptions, whereas low temperatures (≤25 °C) favoured production of desirable green sensory perceptions [149]. The same conclusions were achieved by Kalua et al. [144]. The use of an inert gas in the headspace of malaxer does not significantly affect the production of volatile compounds [141].

#### 3.2.3. Oil Extraction

VOOs are usually obtained by means of pressure and centrifugation systems. Such separation systems differ not only for the physical forces involved in the oil separation, but also in the amount of water used in the process. Generally pressure does not require any addition of water, whereas centrifugation system needs more or less variable amounts of water to allow the separation of the oil from the olive paste. The added water, modifying the original partition equilibria between oil and water present in the olive paste, affects the chemical composition of the oil and, as a consequence, its sensory and healthy characteristics [152,153]. These partition phenomena are of particular importance for the phenolic fraction, essentially formed by hydrophilic compounds.

Phenolics concentration of oils obtained by means of three-phase centrifugation is significantly lower than their level in oils extracted by pressure. In fact the traditional centrifugation needs the addition of a considerable amount of lukewarm water (40–60 L/100 kg of olives) before the centrifugation, to reduce the viscosity of olive pastes. The loss in phenolic compounds extracted by such system, due to a modified distribution for both a dilution effect and a new partition equilibrium [152,153,154,155], has significant repercussions on the induction time and therefore on the shelf-life of VOOs. The sensory analysis highlights a weakening of bitter and pungent attributes as a consequence of the reduced levels of secoiridoids. Moreover, the addition of lukewarm water can explain the decrease of some volatiles, such as hexan-1-ol and *trans*-2-hexen-1-ol, with respect to oils separated by pressure [156,157]. The adoption of centrifugation systems requiring no addition of water (two-phase decanters) or needing small volumes of water, known as water saving decanters (0–30 L/100 kg of olive paste), allows to obtain oils richer in hydrophilic phenols than those obtained by the traditional three-phases centrifuges [48,152,158,159,160,161,162]. Less important changes have been found in volatile composition of oil from two and three phase decanters, due to a lower water solubility of these compounds when compared with phenolics [55]. At sensory levels these oils are more aromatic and more bitter and pungent than those obtained by traditional centrifugation.

## 4. Varietal Characterization

Monovarietal olive oils have some specific characteristics related to the olive variety from which they are obtained. DNA markers have been used to identify cultivars. Montalegre et al. [162] have recently published an extensive review. Food DNA analysis may represent an attractive and alternative choice to the more classical analytical methods, because DNA provides an opportunity for direct comparison of different genetic material [163,164,165,166,167]. However, Claros et al. [168] showed that soil and climate have significant influence on cultivar differentiation during the years and in addition there is evidence that the genetic diversity of olive cultivars is strongly dependent on the region and country of origin [169]. Since the discovery of amplifiable DNA from olive oil, different genetic markers, generally fragments of DNA or specific sequences of DNA bases or nucleotides, have been used to recognize the cultivar employed for oil production [163,165,169,170,171]. The assignment of the cultivar is based on the comparation of genetic markers extracted from olive oil, suitably amplified, with those obtained from olive leaves. As an example, by using genetic markers, Breton et al. [169] identified 66 cultivars from their leaves and then they proposed the use of their database for cultivar identification of monovarietal oils and blended commodities. Recently, Consolandi et al. [172] proved that genetic markers are able to differentiate 49 cultivars, the most common of the Mediterranean basin. In spite of some successes in varietal characterization, ascribable to adoption of genetic markers having a great discriminatory power, this approach shows some limitations due to the difficult extraction of genetic material in sufficient quantity and high quality from oils, since oil DNA is highly degraded. Moreover the reproducibility of the amplification markers depends on the quality of the recovered DNA.

Another approach to achieve the differentiation among olive oils, according to the olive variety, is represented by the use of compositional markers. They are affected by the environmental or technical conditions, and this makes difficult the interpretation of the results obtained. They have been differentiated in major components (fatty acids and triglycerides) and minor components (sterols, phenolic compounds, volatile compounds, pigments, hydrocarbons, and tocopherols), according to their presence in olive oils. Many authors claim that the VOO authentication is possible in relation to cultivar, geographic area [173,174,175], extraction methodologies [149,176,177] by using compositional markers. However, the dependence of the level of many chemical compounds from agronomic and technological factors does not allow to achieve the characterization on the basis of the simple observation of data related to some fractions of VOO composition. Therefore, for the VOO characterization, a great number of variables are needed and data have to be analyzed by statistic techniques or artificial intelligence algorithms. In the application of these statistic methodologies it should be considered the removal of redundant information and the selection of artefacts that can lead to wrong conclusions. In addition, the problem of the characterization is complicated by the fact that data reported in literature are often obtained by using different analytical methodologies and lump them together for characterization. Fatty acids and triacylglycerols, the major components, are the fractions that were mainly used for the varietal characterization of VOOs [161,178,179,180,181,182,183,184,185].

Varietal characterization was also carried out by analyzing only one class of minor compounds [111,175,186,187,188,189,190,191,192,193,194]. Other researchers used more classes to achieve the varietal characterization [38,84,110,195,196,197,198,199,200,201,202]. In a recent investigation [68] volatiles were used for the characterization of oils from 39 cultivars, native of several producer countries and all grown in the same botanical garden. Oils were obtained from fruits harvested at the same ripening degree, processed with the same system and in the same operative conditions. Therefore, as there were no external variables that might unequally affect the enzyme activity of a particular variety, the differences observed in the volatile concentration are related to the variety. Authors first differentiated cultivars according to the content of total volatile compounds, hydrocarbons, aldehydes, alcohols, ketones and esters. A more profound analysis was carried out on C6 compounds because of their importance as green attributes contributors. The application of a cluster analysis to data of C6 compounds gave pieces of evidence of some similarities among the 39 cultivars. Two main groups, in their turn divided in two groups each one, appeared in the dendrogram, so that there were four groups all formed by varieties of different geographical origin (Table 3).

The contribution of volatiles to aroma of the monovarietal oils is different: Luna and co-workers [68] found that thirteen volatiles did not contribute at all because their odour activity values were lower than 1.0, thirteen of them contributed only for a certain number of varieties and only seven concurred to the sensory profile of all cultivars.

The application of solid phase micro-extraction (SPME) to the analysis of VOOs headspace [109] allowed the detection of significant differences in the proportion of C6 esters, *trans*-2-hexenal, *trans*-2-hexen-1-ol and total amount of metabolites from LnA in monovarietal oils obtained from two different areas of Northern Italy. No differences were observed for C5 compounds with respect to the cultivar. Moreover authors gave emphasis to the great differences found in the content of α-copaene, α-farnesene, and hydrocarbons with >20 carbon atoms [111]. These differences prove that hydrocarbons can be possible markers for varietal characterization of VOOs, as suggested by other researchers [91,110,111].

Considerable differences were found in the total amount of volatile compounds, alcohols, aldehydes, ketones, and C6 and C5 compounds of oils obtained from fruits of 18 cultivars grown in the same orchard on the western coast of the Garda Lake (Northern Italy) [203]. On the basis of the differences in volatile compositions and sensory profiles, some of the varieties could be differentiated. Headspace-mass spectrometry (HS-MS) methodology was used to characterize varieties [201,204]; data obtained from this procedure need to be processed by statistical procedures and allow to predict the assignation of monovarietal oils, according to olive varieties and geographical origins, correctly classifying ca. 87% of samples [204].

**Figure 6:**
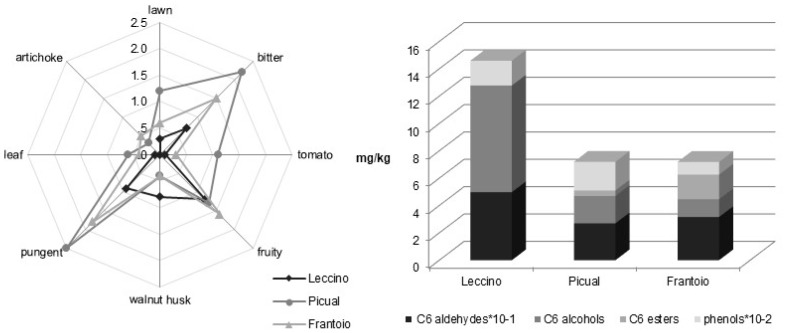

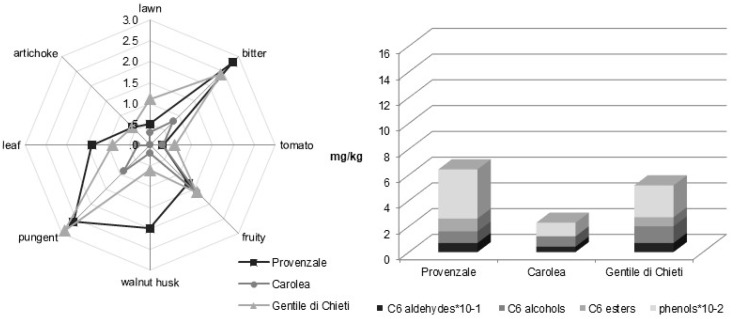
Sensory profiles and concentrations in C6 aldehydes, C6 alcohols and C6 esters and total amount of phenolic compounds of some monovarietal virgin olive oils obtained from fruits harvested at the same ripening degree and processed in the same operative conditions. (Source: Authors; unpublished results).

The different composition in phenolic and volatile compounds from LOX pathway, perceived by consumers as different sensory profiles (Figure 6), can be a useful tool for the varietal characterization. In particular, volatiles arising from LnA LOX pathway have been proposed for the varietal characterization of oils from 20 cultivars [205,206].

The varietal characterization can be made through these metabolites, according to an approach that takes into account the different store of enzymes involved in the LOX pathway of the examined cultivars. This metabolic pathway, having linolenic acid as precursor (Figure 2), mainly accumulates *trans*-2-hexenal and *trans*-2-hexen-1-ol, owing to the isomerisation of *cis*-3-hexenal (A branch), and *cis*-3-hexen-1-ol and *cis*-3-hexenyl acetate, for subsequent enzymatic reduction and esterification of *cis*-3-hexenal (B branch), and involves several enzymes.

The amount of each enzyme is strongly dependent of the enzymatic store of each cultivar, as it is proved by the percent of the corresponding accumulation products (Table 4). The sum of amount of all mentioned metabolites represents the amount of their common precursor *cis*-3-hexenal that, only to a very small extent, does not undergo the activities of isomerase and in some cultivars of alcohol dehydrogenase.

In Table 4 the amount of *trans*-2-hexenal includes also the very small amount of *cis*-3-hexenal since in our gas chromatographic conditions the separation of the two compounds was not complete, because their retention time are very close. The accumulation of *trans*-2-hexenal in all cultivars, except for Moraiolo, evidences that the isomerisation of *cis*-3-hexenal is the dominant process. However, the different amounts of *trans*-2-hexen-1-ol (more than 5%) in some cultivars (Leccino, Dritta, Bosana, Meski, Chetoui) prove a reasonable activity of alcohol dehydrogenases in the reduction of *trans*-2-hexenal, connected with a fair amount of this enzyme in their genetic store.

B branch is active in different way in many of the cultivars examined, namely Carolea, Bosana, Provenzale, Nocellara del Belice, Gentile di Chieti, Maurino, Koroneiki, Pisciottana, Chetoui, Moraiolo. Among them, only Bosana shows a negligible activity of alcohol dehydrogenases and some activity of alcohol acetyl transferases, owing to the accumulation of 5.2% of *cis*-3-hexenyl acetate. In all the other varieties tested, the activity of enzymatic reduction of *cis*-3-hexenal is the most important process of B branch. This last is especially important in Moraiolo, Pisciottana and Maurino, since *cis*-3-hexen-1-ol ranges from 42.4 in Moraiolo to 32.9 in Pisciottana and 20.9 in Maurino, respectively. High levels of alcohol acetyl transferases characterize Koroneiki, Provenzale and Pisciottana.

A branch is the only active in Mastoidis, Coratina, Frantoio, Chemlali, Taggiasca, Canino, and Picual, proving that the activity of isomerase is dominant. The content of *trans*-2-hexenal differentiates the cultivars. However, Mastoidis and Taggiasca show similar content of *trans*-2-hexenal. The percent of *cis*-3-hexen-1-ol and *cis*-3-hexenyl acetate in Taggiasca indicate some activity of enzymes of B branch, practically absent in Mastoidis cultivar. Picual is characterized by some activity of ADH of B branch, proved by a 5% of *cis*-3-hexen-1-ol. Oils from Leccino are characterized only by high activity of isomerases and by a fair activity of ADHs in A branch. Dritta and Bosana, having similar contents of *trans*-2-hexenal and *trans*-2-hexen-1-ol, are differentiated by a more important activity of alcohol acetyl transferases in Bosana, proved by the accumulation of a 5% of *cis*-3-hexenyl acetate.

Both A and B branches are active in oils from Provenzale, Nocellara del Belice, Gentile di Chieti and Maurino. In all these cultivars alcohol dehydrogenases in A branch are practically absent, whereas are active those of B branch that cause the accumulation of *cis*-3-hexen-1-ol. The percent of this metabolite, ranging from about 10% up to 20%, differentiates the cultivars. In addition, Provenzale is characterized by a considerable activity of alcohol acetyl transferases that is negligible in Maurino, whereas play an important role in Koroneiki, Pisciottana, Moraiolo and Chetoui. Therefore it possible to differentiate all cultivars according to the activities of enzymes involved in A and B branches.

Some investigations carried out on oils from Leccino, Coratina and Picual varieties, grown in the botanical garden in two different olive crops from fruits sampled at the same ripening degree and processed in the same conditions, proved that such distribution is the same over the production crop, even if the climatic conditions were very different [206]. On the other hand the same distribution was observed regardless of where the fruit was grown when the fruits show the same ripening degree and are processed in the same conditions: the same results were obtained for oils from Koroneiki and Picual cultivars grown in Central Italy (Pescara) and in Greece (Crete) [206]. Moreover it has been proved that C6 distribution is stable during the ripening process from the half-cherry stage to the black one that is from when the final oil content is reached on. This means its independency from the ripening degree of fruits at least in the usual harvesting time [102]. The independence from where the fruit is grown, the year variability and the ripening degree of olives makes very interesting this approach for varietal characterization and for the control of truthfulness of the cultivar stated on the label. The method is closely dependent on genetic factors, fast, based on a simple head-space analysis and could allow to identify several cultivars, without any application of statistical procedures. The different compositions in volatile compounds, in addition to different content in phenolic compounds, are perceived by consumer as different sensory profiles, as shown in Figure 6.

## 5. Conclusions

Nowadays there is an increasing attention to food. Consumers address their choice to food having special sensory characteristics and able to supply basic nutrients, to improve physical and mental health and to reduce the risk of the most common diseases. High quality VOOs meet all these requirements, being rich in antioxidant compounds having the requested beneficial activities and possessing sensory characteristics that greatly improve the acceptability of food. Sensory profiles of monovarietal VOOs represent an important element able to characterize and differentiate them and, actually, their productions is constantly increasing. Therefore, the main goal of the oil production is to preserve all compounds responsible for sensory notes. The knowledge of the profile of volatiles and phenolics that characterize oil obtained by fruit of a cultivar is, in our opinion, of major importance since it conditions the choice of several parameters affecting the quality of the resulting oil. To achieve the result of an oil with balanced sensory characteristics and highly appreciated by consumers, it is necessary to choose in an appropriate manner factors such as the harvesting time of fruits, the possible amount of irrigation water, the type of system used for the oil extraction, especially the kind of crusher, all greatly dependent by the cultivars.

## Figures and Tables

**Table 1:** Green attributes correlated to volatiles from LOX pathways and to the total amount of phenolic compounds. (Source: Authors; unpublished results).

Sensory Notes	R^2^	Volatile Compounds Positively Related	Volatile Compounds Negatively Related
Bitter	0.80	1-penten-3-one, polyphenols	hexanal, *cis*-3-hexen-1-ol
Pungent	0.80	1-penten-3-one, polyphenols	hexanal, *trans*-2-hexenal
Sweet	0.72	hexanal	*trans*-2-hexenal, *trans*-2-pentenal
Fruity	0.66	*cis*-2-penten-1-ol	*trans*-2-hexen-1-ol, *trans*-2-pentenal, 1-penten-3-one
Leaf	0.65	1-penten-3-one, polyphenols	hexanal
Freshly cut grass	0.57	*trans*-2-hexenal	hexanal
Almond	0.62	*cis*-2-penten-1-ol	*trans*-2-hexenal, 1-penten-3-ol, *cis*-3-hexen-1-ol, polyphenols
Banana	0.60	hexanal, *cis*-3-hexenyl acetate	*trans*-2-pentenal, *trans* -2-hexenal, *cis*-2-penten-1-ol
Walnut husk	0.57	*cis*-3-hexenyl acetate, *trans*-2-pentenal, polyphenols	hexan-1-ol, *cis*-3-hexen-1-ol
Wild flowers	0.56	*trans*-2-hexen-1-ol	hexyl acetate, hexanal
Tomato	0.51	hexan-1-ol, 1-penten-3-one	*trans*-2-hexen-1-ol, hexanal, 1-penten-3-ol

**Table 2:** Levels (mg/kg) of C6 and C5 volatile compounds in some extra virgin monovarietal olive oils. Results are expressed as mean of three samples of each monovarietal olive oil. A = all C6 compounds from LA; B = all C6 compounds from LnA; C = all C5 compounds. (Source: Authors; unpublished results).

Compound	Dritta	Frantoio	Bosana	Moraiolo	Canino	Coratina	Koroneiki
Hexanal	0.7	2.7	1.2	0.1	0.5	0.5	0.8
Hexan-1-ol	0.7	0.3	0.6	0.2	0.2	0.1	0.2
Hexyl acetate	0.2	0.4	0.6	0.1	traces	0.0	0.2
*trans*-2-Hexenal	11.4	38.9	12.1	1.8	30.3	23.8	3.3
*trans*-2-Hexen-1-ol	1.5	0.6	1.5	0.2	0.9	0.6	0.1
*cis*-3-Hexen-1-ol	0.2	0.4	0.3	1.7	0.7	0.3	0.9
*cis*-3-Hexenyl acetate	0.6	1.3	1.1	0.4	0.1	0.1	2.0
2-Pentenal	0.1	0.2	0.2	0.1	0.3	0.3	0.2
1-Penten-3-ol	0.1	0.4	0.2	0.2	0.2	0.4	0.4
1-Penten-3-one	0.2	0.7	0.2	0.2	0.2	0.9	0.8
*cis*-2-Penten-1-ol	traces	0.6	0.5	0.2	0.3	0.4	0.3
Pentene dimers	0.5	0.7	0.6	0.5	1.1	0.6	2.3
Total aldehydes	12.2	42.0	13.5	2.0	31.1	24.6	4.3
Total alcohols	2.5	2.3	3.1	2.5	2.3	1.8	1.9
Total esters	0.8	1.7	1.7	0.5	0.1	0.1	2.2
B/A	8.6	12.1	6.3	10.3	45.7	41.3	5.3
B/C	15.2	17.2	8.8	3.4	15.2	9.5	1.6
Total C6 and C5 compounds	16.2	47.2	19.1	5.7	34.8	28.0	11.5

**Table 3:** Cultivars belonging to the four groups evidenced by HCA, and elements they shared.

Group	Subgroup	Cultivars	Elements Shared
1	a	Arbequina, Coratina, Cima di Bitonto, Chemlal of Kabylie, Frantoio, Manzanilla, Manzanillo Cordobe’s, Mastoides, Moraiolo, Morruda, Negro, Nevado, Nisjot, Santa Caterina, Konservalia	high content of *trans*-2-hexenal; mean content of alcohols and esters; low concentration of hexanal
1	b	Leccino, Lechín, Megaritiki, Ogghiaredda	high content of *trans*-2-hexenal; low concentration of alcohols; high concentration of esters; concentrations of hexanal and alcohols similar
2	a	Cornicabra, Empeltre, Hojiblanca, Imperial, Picual, Memecik, Picholine Marrocaine, Sourani	high concentrations of hexan-1-ol and *cis*-3-hexen-1-ol; very low concentrations of *trans*-2-hexenal and esters; hexanal concentration higher than *trans*-2-hexenal
2	b	Adramytini, Cañivano, Chami, Chetoui, Chorruo, Koroneiki, Nevadillo, Picudo, Redondilla, Tsounati, Verdial de Huévar, Zaity	high content of *trans-*2-hexenal and hexanal, mean content of alcohols; low concentration of and esters

**Table 4:** *trans*-2-Hexenal as mg/kg, and percent distribution of the main metabolites coming from LnA oxidation in some extra virgin monovarietal olive oils. Results are expressed as mean of three samples of each monovarietal olive oil. (Source: the authors, unpublished results).

Cultivar	*trans*-2-Hexenal (mg/kg)	% *trans*-2-Hexenal	% *trans*-2-Hexen1-ol	% *cis*-3-Hexen-1-ol	% *cis*-3-Hexenyl Acetate
Mastoidis	17.1	99.4	0.1	0.5	0.0
Coratina	43.5	97.8	1.5	0.7	0.0
Frantoio	53.4	96.6	1.2	0.7	1.5
Chemlali	14.7	95.6	1.5	1.9	1.1
Taggiasca	17.2	94.9	1.6	1.6	1.9
Canino	30.3	94.8	2.8	2.2	0.2
Picual	23.2	92.6	1.2	5.0	1.2
Leccino	47.3	89.0	10.1	0.9	0.0
Dritta	11.4	84.5	10.9	1.5	3.1
Carolea	7.4	83.4	2.2	14.4	0.0
Bosana	12.1	82.7	10.1	2.0	5.2
Provenzale	5.7	79.4	1.4	9.6	9.6
Nocellara del Belice	6.8	78.4	1.1	15.8	5.0
Gentile di Chieti	6.5	75.1	2.3	18.1	4.5
Maurino	6.3	74.4	2.3	20.9	2.4
Meski	6.2	61.3	37.8	1.0	0.0
Koroneiki	4.6	58.7	3.8	16.3	21.3
Pisciottana	1.1	52.6	4.7	32.9	9.9
Chetoui	3.1	49.5	27.8	17.0	5.6
Moraiolo	1.8	45.6	5.0	42.4	7.0
